# Red-Laser Photodynamic Therapy with Toluidine Blue Gel as an Adjuvant to Topical Antifungal Treatments for Onychomycosis in Patients with Diabetes: A Prospective Case Series

**DOI:** 10.3390/jcm14051588

**Published:** 2025-02-26

**Authors:** David Navarro-Pérez, Sara García-Oreja, Francisco Javier Álvaro-Afonso, Mateo López-Moral, José Luis Lázaro-Martínez, Aroa Tardáguila-García

**Affiliations:** 1 Diabetic Foot Unit, Clínica Universitaria de Podología, Facultad de Enfermería, Fisioterapia y Podología, Instituto de Investigación Sanitaria del Hospital Clínico San Carlos (IdISSC), Universidad Complutense de Madrid, 28040 Madrid, Spain; davinava@ucm.es (D.N.-P.); alvaro@ucm.es (F.J.Á.-A.); matlopez@ucm.es (M.L.-M.); diabetes@ucm.es (J.L.L.-M.); aroa.tardaguila@ucm.es (A.T.-G.); 2 Clínica Universitaria de Podología, Facultad de Enfermería, Fisioterapia y Podología, Instituto de Investigación Sanitaria del Hospital Clínico San Carlos (IdISSC), Universidad Complutense de Madrid, 28040 Madrid, Spain

**Keywords:** photodynamic therapy, topical antifungal, ciclopirox, onychomycosis, diabetes, diabetic foot

## Abstract

**Background**: Systemic therapy is frequently utilized because of its easy accessibility, low cost, and high efficacy. However, it can be linked with systemic adverse effects and drug–drug interactions, especially in immunocompromised and poly-medicated patients. Topical antifungals, associated with a low risk of systemic adverse effects and drug–drug interactions, have emerged as the most suitable treatment option for patients with diabetic foot disease. However, the duration of topical treatment can extend up to 12 months. Consequently, there is a need to bolster these topical treatments with complementary therapies. **Methods**: The current study acquired approval from an ethics committee (code 24/241-E) and Clinical Trials (code NCT06485050). No patients were excluded, irrespective of comorbidities or the severity of onychomycosis. Patients included in the study were administered Ciclopirox 8% (consisting of ethyl acetate, 96% ethanol, ketostearyl alcohol, hydroxypropyl chitosan, and purified water) once daily for 6 months. This was supplemented with photodynamic therapy (three sessions in the first 2 months) using toluidine blue gel and a 635 nm diode laser lasting 10 min, as well as monthly debridement of the nail plate. **Results**: All patients (10/10) included in the study exhibited negative microbiological culture results 6 months after the study began. Of these, 90% (9/10) were clinically cured, and thus, fully cured. No adverse effects or complications secondary to the treatments were observed in any of the cases. The average Onychomycosis Severity Index (OSI) value was initially 18.50 ± 8.947, reduced to 10.30 ± 6.129 at 3 months, and finally fell to 4.10 ± 4.08 at the end of the treatment. **Conclusions**: The current study demonstrated the clinical improvement, mycological cure, effectiveness, and safety of combination therapy of ciclopirox 8% and photodynamic therapy over 6 months.

## 1. Background

One-third of individuals with diabetes mellitus (DM) also present with onychomycosis (ONM), primarily due to a weakened immune system that is caused by prolonged hyperglycemia [1,2]. The main clinical signs of onychomycosis include thickening, hyperkeratosis and subungual detritus, chromonychia, onycholysis, dermatophytomas, and there are several types, such as distal and lateral subungual onychomycosis, proximal subungual onychomycosis, superficial white onychomycosis, and total dystrophic onychomycosis [3,4,5,6]. For patients with diabetic foot, total dystrophic ONM poses a risk owing to the presence of nail thickening and subungual hyperkeratosis, which can lead to periungual lesions or injuries in the nail bed, thereby facilitating the entry of other microorganisms. This can lead to infections and ulcers [7,8].

Therefore, it is crucial to treat ONM in this patient profile. Oral treatments, such as terbinafine, itraconazole, and fluconazole, are the gold standard for ONM treatment, particularly in severe cases, which exhibit 40–80% cure rates [9]. However, while these treatments have been deemed safe for patients with DM and without significant risks or interactions, the reality may be different. A recent systematic review analyzed studies involving diabetic patients with onychomycosis treated with oral antifungals and found virtually no interactions, deeming the treatments safe [10]. However, considering the exclusion criteria of these studies—which include peripheral vascular disease (PVD), diabetic neuropathy, liver dysfunction, inadequate liver and/or renal control, use of immunosuppressants, severe onychomycosis, ischemic pain, renal failure, uncontrolled diabetes, corticosteroid use, nephropathy, and blood disorders—these findings might not apply to all diabetic individuals. Moreover, these treatments can potentially be associated with systemic adverse events and drug–drug interactions. Thus, they are not recommended for diabetic, immunocompromised, and frequently poly-medicated individuals, even though these individuals have a high risk of fungal infection [2,8,10,11,12,13].

Other lines of treatment may include topical antifungals such as allylamines, azoles, hydroxypyridones, morpholine derivatives, and benzoxaborole (e.g., tavaborole) [8]. Topical antifungals or lacquers are associated with a low risk of systemic adverse events and drug–drug interactions, making them the most suitable treatment for patients with diabetic foot [2,9,14]. However, they necessitate very long treatment durations, are generally applied daily for 12 months to allow the normal nail to grow and replace the areas damaged by the infection, have a lower cure rate, and are not recommended for severe onychomycosis [8,9,15].

On the other hand, physical therapies such as laser therapy and photodynamic therapy [9,12] are available. Laser therapy is contraindicated for patients with diabetes due to the risk of burns, which can lead to ulcers [8,12]. Photodynamic therapy triggers fungal apoptosis by shining specific wavelengths of light onto fungal cells containing photosensitizers [12,16]. These activated photosensitizers generate highly reactive oxygen species, thereby leading to fungal apoptosis [16]. Photosensitizers like 5-aminolevulinic acid, methyl aminolevulinate, and methylene blue have successfully been used in the treatment of patients with onychomycosis [12]. Negative culture and/or negative microscopy results were found in 67% of patients in six photodynamic therapy studies conducted in recent years [17,18,19,20,21,22]. A 2015 review on the effectiveness of photodynamic therapy in treating onychomycosis showed that photosensitizers like methylene blue and 5-aminolevulinic acid yielded cure rates of 80% and 43% respectively [23]. A drawback of photodynamic therapy is that the application of the photosensitizer typically needs to be applied hours before the session and thus requires patient care at home [17].

Given the mentioned limitations, there is a need to improve routine clinical practice’s topical treatments, especially for patients with diabetes, diabetic foot, elderly patients, or patients with other comorbidities. This can be accomplished by incorporating coadjuvant therapies such as photodynamic therapy with photosensitizers, which are applicable in the consulting room. This treatment can be used for onychomycosis in patients with poorly managed diabetes without excluding any comorbidities. The goal is to observe the efficacy and safety rates of pairing photodynamic therapy with the normal antifungal treatment. Additionally, we wish to determine if three sessions of photodynamic therapy combined with topical therapy, spread over 6 months, can successfully treat onychomycosis clinically, mycologically, and wholly. Moreover, we aim to analyze if the fungal agent causing the onychomycosis, the type of onychomycosis, and its severity influence the treatment’s response.

## 2. Materials and Methods

### 2.1. Participants

This study was conducted following the Declaration of Helsinki and the current national legislation that governs research involving patients [24]. Before participating in the study, the patients signed informed consent forms, and the study has been approved by an ethics committee (code 24/241-E; 17 April 2024; CEIm Hospital Clínico San Carlos). Additionally, it is registered in Clinical Trials (Code: NCT06485050). A prospective case series was carried out between April and November 2024.

Patients over 18 years of age, who had received diagnostic confirmation via microbiological culture and PCR in a specialized diabetic foot unit, were included [25]. The sample selection required sufficiently large portions of the nail to allow for both diagnostic procedures [25]. The samples were examined by microbiological culturing in Sabourad dextrose agar from 1 to 3 weeks in an external laboratory. PCR was performed based on the protocols of previous studies [25]. After 1–3 weeks, the laboratory gave us the results.

Patients who had undergone antifungal treatment (topical or systemic) in the previous month, had psoriasis, lichen planus or subungual tumours, and those unable to apply topical treatment due to problems with self-care, hygiene, and limited mobility were excluded.

At the commencement of the study, we had a total of 59 patients with DM and onychomycosis available for treatment. Of these, 32 were excluded because of limited mobility and health issues, while another 17 were also excluded due to insufficient self-care. Subsequently, ten patients were included in the study.

### 2.2. Intervention and Follow-Up of Patients

At the first visit of the study, the patient was prescribed a topical antifungal used in routine clinical practice: Ciclopirox 8% ethyl acetate, ethanol (96%), ketostearyl alcohol, hydroxypropyl chitosan, and purified water (Ony-tec^®^, Almirall S.A, Barcelona, Spain). A daily application was recommended. Moreover, all patients received the following hygiene recommendations: daily washing with acidic pH soap (5.5), thorough drying of the entire foot, daily disinfection of footwear after use, discarding very old and potentially contaminated footwear, avoiding the sharing of footwear, minimizing microtrauma to nails through a good footwear fit, and avoiding potential exposure areas by not going barefoot in places like public showers and swimming pools.

Follow-ups by the same investigator were carried out every 2 weeks during the first 2 months, with photodynamic therapy applied during the second, third, and fourth visits. A check-up took place 2 weeks after the last application of photodynamic therapy, followed by monthly visits for the remainder of the study.

At each visit and subsequent check-ups, the nail plate and both peri- and subungual hyperkeratotic tissue were debrided and trimmed to enhance the effects of the treatment. The procedure for the photodynamic therapy application proceeded as follows: (1) mechanical debridement of the nail; (2) disinfection of the nail with alcohol; (3) application of a topical photosensitizer (Toluidine blue gel), which involved keeping the nail covered for 5 min to prevent premature chemical activation due to exposure to natural light; and (4) an application of 635 ± 10 nm red diode laser for 10 min to activate the topical photosensitizer using the Rapido Podia Diode Laser^®^ (Medency Srl, Vicenza, Italy). The delivery system involved the DIRECTO^®^ collimated handpiece (Medency Srl, Vicenza, Italy), which has a 1 cm^2^ spot.

After the 6-month treatment was completed, a culture sample was taken 1 month following the final application of the topical antifungal. This was carried out to assess the cure rate and to validate mycological and complete cures when a negative result is obtained [25]. The definition of clinical cure fluctuates across studies, ranging from 0% nail plate involvement to more than 50% clinical improvement [12,26]. Mycological cure is confirmed by the negative outcome of microbiological tests, and a complete cure comprises clinical cure complemented by a negative laboratory test result [12,26]. Thus, the objective of treatment will be to achieve a complete cure, which would entail both eradicating the infectious organism (mycological cure) and restoring the affected structure to its original appearance (clinical cure) [11].

In addition to corresponding debridement and treatment, several photographs were taken of the study nail during each visit for photographic documentation. These photographs were received by the evaluator at the end of each visit to facilitate a detailed photographic follow-up. They were then used to assess the severity of the onychomycosis using the Onychomycosis Severity Index (OSI) at the start of the study, after 3 months, and after the end of the study [27].

### 2.3. Blinding

The severity of the onychomycosis was evaluated by a second investigating podiatrist who assessed the clinical cure in consultation, at the beginning, and at the end of treatment, the OSI of each of the visits, and the microbiological cure through the results obtained by the microbiological study performed by an external microbiology department [25,27].

### 2.4. Statistical Analysis

The statistical analysis was conducted using SPSS for Windows, version 22.0 (SPSS, Inc., Chicago, IL, USA).

The sample was characterized using tables and graphs that displayed qualitative variables as frequency and percentage, and quantitative variables as mean and standard deviation.

The primary outcome measure was defined as the complete clinical and mycological cure rate.

## 3. Results

A total of ten patients were enrolled in the study, of which seven were male (70%) and three were female (30%). The average age of all patients was 66.10 ± 10.29. Sixty percent (6/10) of the patients suffered from DM2, 40% (4/10) from DM1, and 50% (5/10) had diabetic foot. The other comorbidities are outlined in Table 1.

Regarding pathogens, the most common infections were mixed infections, followed by yeasts, molds, and dermatophytes, as shown in Table 2.

In the study, all of the included patients (100%, 10/10) demonstrated negative microbiological culture results 6 months from the commencement of the study. Ninety percent (9/10) of these individuals achieved a total clinical cure. On the other hand, 10% (1/10) maintained clinical signs of onychomycosis at the study’s conclusion, according to the second evaluator.

**Figure 1:**
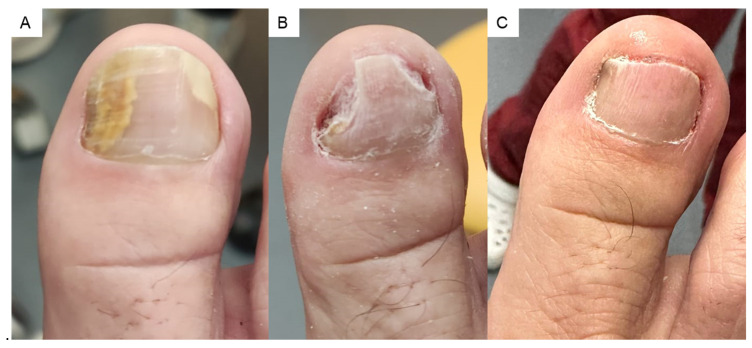
Follow-up of onychomycosis. (**A**) Consultation where the sample is taken for microbiological culture. (**B**) Mechanical debridement of dermatophytoma and onycholysis. (**C**) Nail appearance after 6 months of treatment.

During the 6-month follow-up, no adverse effects or complications were observed as a result of the treatments in any of the cases (Figure 1).

Table 3 and Table 4 display the patients’ initial, 3-month, and final OSI results and type of onychomycosis, indicating an improvement between the initial and final OSI in all cases.

The average OSI was 18.50 ± 8.95 at the initial treatment, 10.30 ± 6.13 at the three-month mark, and 4.10 ± 4.08 at the final treatment.

## 4. Discussion

In this study involving ten patients with DM and ONM, we observed a 100% mycological cure rate with no adverse effects over 6 months, regardless of the severity of ONM, its type, and the pathogen causing the infection.

Additionally, even if not all patients achieved a mycological cure, there was a noticeable clinical improvement in the condition of the nails. This indicates that the combination of both treatments is an effective course of action for mild, moderate, and severe ONM, especially for patients for whom other treatments are not recommended.

The mechanical debridement performed at each visit is also important to facilitate the effectiveness of the treatment [28] (Figure 1).

Previous studies have defined oral and topical antifungal treatments such as terbinafine, itraconazole, ciclopirox, and efinaconazole as safe and effective for patients with DM [10]. However, all these studies excluded patients with severe comorbidities like renal and hepatic pathology, diabetic foot, PVD, neuropathy, history of ulcers and amputations, and uncontrolled diabetes, as well as those with moderate or severe ONM [10].

Most drugs utilized in dermatology, including systemic antifungals, are metabolized in the liver. The CYP 3A4 isoform is the most prevalent cytochrome isoform, accounting for 60% to 70% of hepatic and enterocyte cytochrome enzymes [10,29,30]. Consequently, liver disease, which is frequent in these patients, would serve as a contraindication to systemic treatment [29,30]. Moreover, most of these patients are provided with multiple treatments, and many drugs interact with oral antifungals such as oral antidiabetics and anticoagulants. There are also specific interactions with certain antifungals like statin-type lipid-lowering drugs and itraconazole [29,30,31]. Hence, it is essential to conduct studies that accommodate these variables.

As a result, it is imperative to explore alternative treatment protocols for these patients, as demonstrated by the approach we used in the current study. This method resulted in a mycological cure rate of 100% and a complete cure rate of 90%.

Conversely, until now, clinical trials involving various topical antifungal treatments or external therapies such as laser have not included immunocompromised patients or patients with DM. To the best of our knowledge, this is the first study to encompass patients with all comorbidities, and mild, moderate, and severe ONM. The topical antifungal treatment using nail lacquers exhibits low and highly varied efficacy rates between studies, as well as requiring extended application times [8,12]. Past studies of photodynamic therapy involving various photosensitizing gels, such as aluminum-phthalocyanine chloride nanoemulsions or methylene blue, have indicated cure rates of 30–70% when applied to healthy adults with onychomycosis [32,33,34,35]. In this study, not only did we achieve a higher rate of cure, but the photosensitizer gel also required a significantly shorter application time. Usually, the photosensitizer needs to be applied several hours before the session, requiring the patient to perform home care, whereas the application of the toluidine blue gel only takes 5 min before the photodynamic therapy [17].

In our study, 90% achieved a complete cure and all participants achieved a mycological cure despite not finishing the recommended application time for the antifungal product (9–12 months). The product used was Ciclopirox hydroxypropyl chitosan [36]. This suggests that combining this product with photodynamic therapy enhances the effects and shortens treatment durations.

In addition, the advantages of topical treatments in patients with diabetes, older patients, or patients with other comorbidities are a lower probability of systemic adverse effects and the fact that they do not interact with drugs that patients may take for other pathologies or pose a risk to the liver or kidneys as oral treatments do [8,10]. Therefore, in this patient profile, the use of topical therapies, or in this case, a combination of two of them, is recommended [8,10]. However, other therapies, such as laser therapy, are not recommended in this patient profile due to the injuries they can cause and their consequences, despite the fact that they have been shown to be effective in a population without associated comorbidities [37]. Some alternative therapies currently under investigation include the use of essential oils (EOs), such as *Ageratina pichinchensis*, *Thymus vulgaris*, *Cinnamomum zeylanicum*, or *Melaleuca alternifolia* [38,39,40,41,42]. EOs may offer significant advantages, particularly in patients with diabetes mellitus, as they do not cause drug–drug interactions or hepatotoxicity. However, they should be studied further, as some cases of allergies and skin irritation have been reported [38,39,40,41,42].

The limitations of this study include its small sample size, classifying it as a pilot study. The fact that it was a single-center study also contributes to its constraints. Additionally, the follow-up time was limited to 6 months, despite the indication for topical treatment spanning 9–12 months. Furthermore, we did not assess recurrence in either the short or long term. Finally, the absence of a control group hinders comparisons and the execution of more advanced statistical analyses.

Future studies could benefit from enlarging the sample size, incorporating a control group for randomized clinical trials, and extending the follow-up period. Doing so would enhance the reliability of results and conclusions.

In conclusion, the current study demonstrated the clinical improvement, mycological cure, efficacy, and safety of using ciclopirox 8% in conjunction with photodynamic therapy utilizing toluidine blue gel. This treatment, administered over 6 months, proved viable for managing ONM in patients who are dealing with diabetes and diabetic foot.

## Figures and Tables

**Table 1:** Characteristics, comorbidities, and risk factors of the study population.

	n (%)
HT	8 (80)
Cholesterol	7 (70)
Neuropathy	5 (50)
Retinopathy	1 (10)
Nephropathy	3 (30)
Cardiovascular history	2 (20)
Endocrine control	8 (80)
Arthritis	4 (40)
Arthrosis	5 (50)
PVD	3 (30)
Diabetic foot	5 (50)
History of ulcers	5 (50)
History of minor amputations	4 (40)
Anti-aggregants	4 (40)
OAC	2 (20)
Cholesterol medication	7 (70)
HT medication	8 (80)
Antidiabetics	
OAD	2 (20)
Insulin	1 (10)
OAD + insulin	7 (70)
Polymedicated	8 (80)

HT, hypertension; PVD, peripheral vascular disease; OAC, anticoagulant; OAD, oral antidiabetic.

**Table 2:** Clinical and pathogenic signs.

	n (%)
Localisation	
Hallux right foot	5 (50)
Hallux left foot	5 (50)
Years of development	
<1 year	1 (10)
>1 year	9 (90)
Nail thickening	6 (60)
Subungual hyperkeratosis	6 (60)
Chromonychia	9 (90)
Onycholysis	6 (60)
Dermatophytoma	2 (20)
Detritus	5 (50)
Longitudinal striae	4 (40)
Type of fungi	
Dermatophyte	1 (10) (Not detected)
Mould	1 (10) (*Curvularia* sp.)
Yeast	3 (30) (*Candida* sp.)
Mixed	5 (50) *

* Mixed: *Candida* sp. + dermaphyte (2); *Candida* sp. + *Fusarium* sp.; *Candida* sp. + Trichophyton Mentagrophytes (2).

**Table 3:** Onychomycosis Severity Index (OSI) at the start of treatment, at three months, and at the end of treatment.

	Initial OSI (%)	3 Months OSI (%)	6 Months OSI (%)
Mild	1 (10)	3 (30)	8 (80)
Moderate	1 (10)	4 (40)	1 (10)
Severe	8 (80)	3 (30)	1 (10)

**Table 4:** Type of onychomycosis at the beginning of treatment, at 3 months, and at 6 months.

	Initial	3 Months	6 Months
Distal	1 (10)	3 (30)	0 (0)
Distal-Lateral	4 (40)	3 (30)	0 (0)
Superficial	1 (10)	1 (10)	1 (10)
Dystrophic	4 (40)	3 (30)	0 (0)
Clinical cure	0	0	9 (90)

## Data Availability

The data that support the findings of this study are available from the corresponding author upon reasonable request.
